# Biased GPCR signaling by the native parathyroid hormone–related protein 1 to 141 relative to its N-terminal fragment 1 to 36

**DOI:** 10.1016/j.jbc.2022.102332

**Published:** 2022-08-04

**Authors:** Karina A. Peña, Alex D. White, Sofya Savransky, Ignacio Portales Castillo, Frédéric G. Jean-Alphonse, Thomas J. Gardella, Ieva Sutkeviciute, Jean-Pierre Vilardaga

**Affiliations:** 1 School of Medicine, Department of Pharmacology and Chemical Biology, University of Pittsburgh, Pittsburgh, USA; 2 Graduate Program in Molecular Pharmacology, University of Pittsburgh School of Medicine, Pittsburgh, USA; 3 Endocrine Unit, Massachusetts General Hospital and Harvard Medical School, Boston, Massachusetts, USA

**Keywords:** parathyroid hormone–related protein, PTH receptor, GPCR, cAMP signaling, location bias signaling, βarr, β-arrestin, GAG, glycosaminoglycan, PTH, parathyroid hormone, PTHR, PTH receptor

## Abstract

The parathyroid hormone (PTH)–related protein (PTHrP) is indispensable for the development of mammary glands, placental calcium ion transport, tooth eruption, bone formation and bone remodeling, and causes hypercalcemia in patients with malignancy. Although mature forms of PTHrP in the body consist of splice variants of 139, 141, and 173 amino acids, our current understanding on how endogenous PTHrP transduces signals through its cognate G-protein coupled receptor (GPCR), the PTH type 1 receptor (PTHR), is largely derived from studies done with its N-terminal fragment, PTHrP_1-36_. Here, we demonstrate using various fluorescence imaging approaches at the single cell level to measure kinetics of (i) receptor activation, (ii) receptor signaling *via* Gs and Gq, and (iii) receptor internalization and recycling that the native PTHrP_1-141_ displays biased agonist signaling properties that are not mimicked by PTHrP_1-36_. Although PTHrP_1–36_ induces transient cAMP production, acute intracellular Ca^2+^ (iCa^2+^) release and β-arrestin recruitment mediated by ligand–PTHR interactions at the plasma membrane, PTHrP_1-141_ triggers sustained cAMP signaling from the plasma membrane and fails to stimulate iCa^2+^ release and recruit β-arrestin. Furthermore, we show that the molecular basis for biased signaling differences between PTHrP_1-36_ and properties of native PTHrP_1-141_ are caused by the stabilization of a singular PTHR conformation and PTHrP_1-141_ sensitivity to heparin, a sulfated glycosaminoglycan. Taken together, our results contribute to a better understanding of the biased signaling process of a native protein hormone acting in conjunction with a GPCR.

Upon its activation, the parathyroid hormone (PTH) receptor (PTHR) triggers both G_s_/cAMP/PKA and G_q_/Ca^2+^/PKC signaling cascades. Developments in recording GPCR-signaling cascade in individual cells in real time using optical approaches during the decade of the ‘00s (1, 2) have revealed that PTH_1-34_ and PTHrP_1-36_ differ markedly by the duration and cellular localization of the cAMP response (3). Brief stimulation with PTHrP_1-36_ induces only transient cAMP production from the cell surface that is rapidly desensitized upon recruitment of β-arrestins (βarrs), cytosolic adapter proteins that canonically act to occlude further G protein coupling and promote translocation of the ligand–receptor complex from the cell surface to early endosomes. In contrast, PTH_1-34_ causes an additional sustained phase of cAMP generation *via* PTH–PTHR–βarr complexes that remain active in early endosomes. Thus, this distinction in the spatiotemporal cAMP profiles of PTH and PTHrP was proposed to be the underlying determinant responsible for their biological specificity.

Mature forms of PTH and PTHrP are originally synthesized and secreted as 84 aa and 141 aa proteins, respectively. Early reports demonstrating that their respective N-terminal part, PTH_1-34_ and PTHrP_1-36_, retain their full capacity to stimulate adenylyl cyclase in cAMP accumulation assays led to the utilization of these N-terminal fragments in most studies. Indeed, it was PTH_1-34_ and PTHrP_1-36_ that were used in the aforementioned work that revealed differences in the time courses and subcellular locations of cAMP production by these two peptides. In contrast to these earlier findings of transient signaling by PTHrP_1-36_, a recent publication proposed sustained endosomal cAMP generation induced by full-length PTHrP_1-141_ (4). The authors employed a combination of radioimmunoassays and chemical inhibitors to suggest that PTHrP_1-141_ induces prolonged cAMP signaling in an endocytosis-dependent manner analogous to that observed for PTH_1-34_; however, cAMP experiments were performed in the presence of phosphodiesterase inhibition, which provided a measure of the cumulative levels of cAMP produced during a defined time interval, as opposed to the dynamic levels of cAMP that result from the net effects of its production and breakdown. Furthermore, the chemical compounds utilized to inhibit endocytosis generated inconsistent results with experiments showing no reduction of sustained cAMP responses induced by PTHrP_1-141_ or PTH_1-34_, while others showed only reduction for PTHrP_1-141_ but not for PTH_1-34_. Reduction of PTH_1-34_-induced sustained cAMP response by blocking receptor endocytosis is expected given this ligand’s established ability to signal *via* internalized PTHR from early endosomes (3, 5, 6, 7, 8, 9). These considerations motivated the necessity to implement alternative methods that permit analysis of real-time cAMP response kinetics in real time in single cells. The results unveil the mechanism by which PTHrP_1-141_ engages in sustained signaling and how this differs from the transient effects observed with the N-terminal fragment PTHrP_1-36_.

## Results and discussion

**Figure 1:**
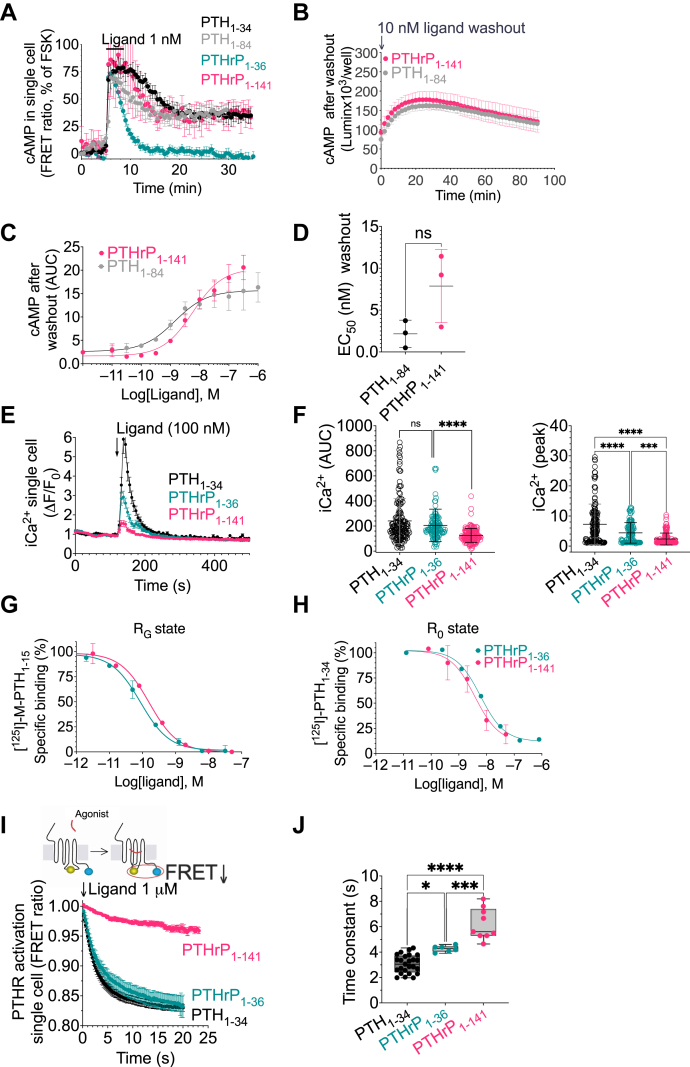
**Signaling properties of PTHrP**_**1-141**_**.***A*, time courses of cAMP in single HEK293 cells stimulated for 30 s with 1 nM ligands. Data are the mean ± SEM of *n* = 37 (PTHrP_1-36_), *n* = 21 (PTHrP_1-141_), *n* = 6 (PTH_1-84_), and *n* = 7 (PTH_1-34_) cells. *B*, time courses of cAMP in HEK-293 cells after washout of ligands measured by Glo-sensor assay. Data are the mean ± SEM of *n* = 3 experiments. *C* and *D*, relationship between cAMP responses in HEK-293 cells after washout of a range of ligand concentrations (*C*) and corresponding EC_50_ values (*D*). Data represent the integrated response determined by measuring the area under the curve of experiments shown in panel (*B*) and are the mean ± SD of *n* = 3 experiments. ns, not significant with *p* = 0.097 by *t* test. *E*, intracellular Ca^2+^ mobilization measurements in single HEK-293 cells stably expressing PTHR. Data are the mean ± SEM for *n* = 44 (PTH_1-34_), *n* = 42 (PTHrP_1-36_), and *n* = 48 (PTHrP_1-141_) cells. *F*, scatter plots with the mean ± SD of data shown in panel (*C*). ∗∗∗∗*p* < 0.0001 determined by one-way ANOVA with Tukey–Kramer post hoc test. (*G* and *H*) competition binding at equilibrium with [^125^I]-PTH_1-15_ and [^125^I]-PTH_1-34_ as radioligands to detect the R^G^ (*E*) and R^0^ (*F*) states of PTHR, respectively. Data are mean ± SD from *N* = 2 independent experiments with duplicate wells for each concentration. *I* and *J*, kinetics of PTHR activation. Normalized activation kinetics of PTHR determined by FRET ratio changes from HEK293 cells expressing the receptor sensor (scheme) (*G*), and time constant (τ) of PTHR activation determined by fitting curves in panel (*A*) to a monoexponential decrease (*H*). Mean ± SEM of *n* = 25 (PTH_1-34_), *n* = 6 (PTHrP_1-36_), and *n* = 9 (PTHrP_1-141_) cells. ∗*p* = 0.0114, ∗∗∗*p* = 0.0003, and ∗∗∗∗*p* < 0.0001 determined by one-way ANOVA with Tukey–Kramer post hoc test. PTH, parathyroid hormone; PTHR, PTH receptor.

We utilized FRET to record real-time courses of cAMP production in single HEK293 cell stably expressing PTHR (HEK-PTHR). We found that brief stimulation with PTHrP_1-141_ induced a sustained cAMP response that was similar in both magnitude and duration to that induced by PTH_1-84_ or PTH_1-34_ and clearly distinct from the short-lived cAMP response mediated by PTHrP_1-36_ (Figs. 1*A* and S1). We next applied Glo-sensor cAMP accumulation assays to verify that time courses of sustained cAMP production mediated by the two native hormones, PTH_1-84_ and PTHrP_1-141_, were similar (Fig. 1*B*) and without a significant difference in the hormone concentration dependence (Fig. 1, *C* and *D*). We observed a striking inability of PTHrP_1-141_ to efficiently induce the release of intracellular calcium (iCa^2+^) from the endoplasmic reticulum (Fig. 1, *E* and *F*), indicating defective Gq activation by PTHrP_1-141_. We have previously shown that Gq activation is required for endosomal cAMP generation by PTH_1-34_ (9), suggesting a differential location of cAMP generation by this ligand. Moreover, the molecular basis for the failure of PTHrP_1-141_ to mimic cAMP and iCa^2+^ signaling responses mediated by PTHrP_1-36_ were unlikely to be caused by different binding affinities to either G protein coupled (R_G_) or uncoupled (R_0_) states of PTHR (Fig. 1, *G* and *H*) but were rather due to the stabilization of a distinct receptor conformation. We tested this theory by using cells expressing a FRET-based PTHR sensor (scheme in Fig. 1*I*). Time-resolved determination of intramolecular FRET changes recorded from single cells allows the analysis of the kinetics of receptor activation in response to ligand binding (1). A decrease in FRET mediated by an agonist reflects receptor switching from an inactive to an active conformation, and distinct time-constants of receptor activation measured for a saturating concentration of agonists indicate the stabilization of distinct signaling receptor conformations (1, 3, 10). As expected, perfusion of a saturating concentration of PTH_1-34_, PTHrP_1-36_, or PTHrP_1-141_ to individual cells triggered a decrease in FRET; however, the significantly distinct time constants (τ) for receptor activation indicated the stabilization of distinct PTHR conformations (Fig. 1, *I* and *J*).

**Figure 2:**
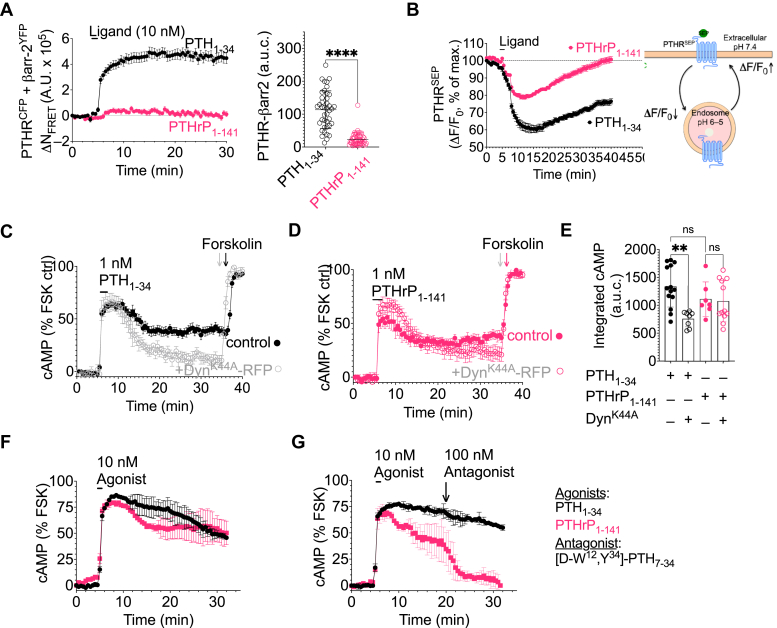
**Endosomal cAMP signaling by PTHrP**_**1-141**_**.***A*, time course of β-arrestin interaction with PTHR in HEK293 cells transiently expressing PTHR^CFP^ and βarr-2^YFP^ treated with 10 nM PTH_1-34_ (*black*) or PTHrP_1-141_ (*red*) for 30 s. Data are the mean ± SEM for *n* = 40 (PTH_1-34_) and *n* = 49 (PTHrP_1-141_) cells. The scatter plot shows the mean ± SD of the integrated response determined by measuring the area under the curve (a.u.c.) ∗∗∗∗*p* < 0.0001 by *t* test. *B*, time courses of internalization and recycling of PTHR tagged with super-ecliptic pHluorin (PTHR^SEP^) in response to 100 nM ligand measured by time-lapse confocal microscopy in single cells. The schematic illustrates the measured values. Data are mean ± SEM from *n* = 12 (PTH_1-34_) and *n* = 51 (PTHrP_1-141_) cells. *C–E*, time courses of cAMP in single HEK-293 PTHR cells transiently expressing with DynK44A^RFP^ compared to control in response to PTH_1-34_ (*C*) and PTHrP_1-141_ (*D*). Data are the mean ± SEM for *n* = 14 cells (PTH_1-34_ control), *n* = 9 cells (PTH_1-34_ Dyn^K44A^), *n* = 8 (PTHrP_1-141_ control), and *n* = 12 (PTHrP_1-141_ Dyn^K44A^) cells. *E*, the scatter plot represents the area under the curve (a.u.c.) corresponding to individual values and the mean ± SD. ∗∗*p* = 0.0017 determined by one-way ANOVA with Tukey–Kramer post hoc test. *F*, time courses of cAMP in single HEK-293 PTHR cells stimulated for 30 s with 10 nM PTH_1-34_ (*black*) or PTHrP_1-141_. Data are the mean ± SEM of *n* = 32 (PTH_1-34_) cells and *n* = 46 (PTHrP_1-141_) cells. *G*, similar experiments as in panel (*E*) with addition of cell-impermeable PTHR antagonist 15 min after washout of PTH_1-34_ or PTHrP_1-141_. Data are the mean ± SEM of *n* = 50 (PTH_1-34_) cells and *n* = 37 (PTHrP_1-141_) cells. PTH, parathyroid hormone; PTHR, PTH receptor.

To assess the role of βarr recruitment, we measured PTHR–βarr interactions *via* FRET in cells transiently expressing PTHR^CFP^ and βarr-2^YFP^. The βarr2 isoform was randomly selected, given that earlier studies demonstrated that PTH_1-34_ and PTHrP_1-36_ displayed equal potencies (EC_50_ values) for recruitment of both β-arr1 and β-arr2 (8, 11, 12). Consistent with previous studies, addition of PTH_1-34_ resulted in significant association of βarr with the receptor that was stably maintained following ligand washout (Fig. 2*A*). In contrast, analogous experiments using PTHrP_1-141_ failed to promote this interaction (Fig. 2*A*), suggesting that the sustained signaling observed for PTHrP_1-141_ occurs in a βarr-independent manner. This finding led us to test the role of receptor internalization, a key step in PTHR endosomal signaling. Measurements of receptor internalization and recycling in single cells stably expressing PTHR^SEP^, the PTHR N-terminally tagged with a pH-sensitive GFP (super-ecliptic pHluorin SEP) that exhibits fluorescence intensity reduction in the acidic environment encountered in endosomes (scheme in Fig. 2*B*), showed reduced internalization and faster recycling in response to PTHrP_1-141_ or PTHrP_1-36_ when compared to PTH_1-34_ (Figs. 2*B* and S2). We next determined whether internalized PTHrP_1-141_-PTHR can signal *via* cAMP. We have previously shown that expression of a dominant-negative dynamin mutant, DynK44A, effectively blocks translocation of PTH–PTHR complexes from the cell surface and blunts the sustained phase of cAMP generation without affecting the forskolin response (3, 8). Accordingly, we compared the cAMP response following brief stimulation with PTHrP_1-141_ in HEK-PTHR control cells and those transiently expressing DynK44A fused to a red fluorescent protein (DynK44A^RFP^) (Fig. 2*C*). Strikingly, blockade of receptor internalization significantly reduced the magnitude and duration of cAMP production by PTH_1-34_ (Fig. 2, *C* and *E*) but had no effect on cAMP mediated by PTHrP_1-141_ (Fig. 2, *D* and *E*), indicating that native PTHrP does not promote sustained signaling in an endocytosis-dependent manner. We recently reported on the development of G_s_-biased PTH analogs that stimulate sustained cAMP production exclusively from the cell surface due to retention of active ligand–receptor complexes at the cell surface. This was experimentally confirmed *via* cAMP time courses using a cell-impermeable PTHR antagonist, which completely abolished the sustained phase of cAMP generation for G_S_-biased peptides but not for PTH_1-34_, consistent with its ability to signal from intracellular compartments (8). We thus utilized this same approach to test whether PTHrP_1-141_ likewise induces prolonged cAMP signaling *via* ligand–receptor complexes that are localized to the cell surface. Indeed, addition of the cell-impermeable antagonist at 15 min following agonist washout rapidly reduced cAMP levels to baseline in cells treated with PTHrP_1-141_ but had no effect in those stimulated with PTH_1-34_ (Fig. 2, *F* and *G*). These findings demonstrate that PTHrP_1-141_ promotes sustained cAMP responses *via* active ligand–receptor complexes localized to the cell surface, which appear inconsistent with experiments showing receptor internalization.

**Figure 3:**
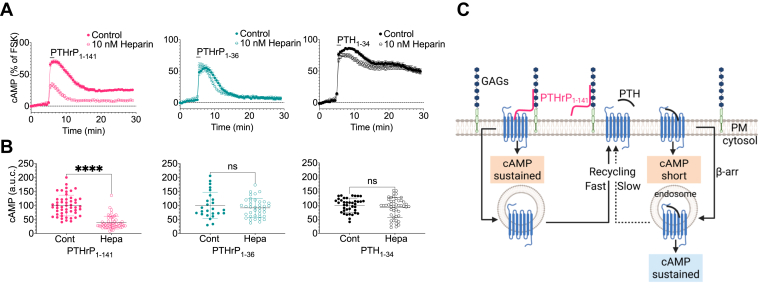
**Effect of heparin on cAMP production.***A*, cAMP time courses in single HEK-293 PTHR cells in response to 1 nM ligands preincubated with 10 nM heparin. Data are the mean ± SEM of *n* = 51 (control) and *n* = 52 (heparin) cells for PTHrP_1-141_; *n* = 25 (control) and *n* = 39 (heparin) cells for PTHrP_1-36_; *n* = 39 (control) and *n* =46 (heparin) cells for PTH_1-34_. The statistical analysis is in Table 1. *B*, corresponding scatter plots representing the area under the curve (a.u.c) of individual values from (*A*). *∗∗∗∗p* < 0.0001 determined by *t* test. *C*, proposed mechanism for location-biased signaling of native PTHrP_1-141_. The continuous cAMP signaling mediated by PTHrP_1-141_ can be controlled by plasma membrane–anchored glycosaminoglycans that hypothetically retain PTHrP_1-141_ at the cell surface thus permitting reactivation of recycled receptors. Created with BioRender.com. PTH, parathyroid hormone; PTHR, PTH receptor.

To reconcile this apparent incompatibility, we hypothesized that the highly positively charged domain of PTHrP_1-141_ (^88^KKKKGKPGKRKEQEKKKRRTR^108^), not present in PTHrP_1-36_ or PTH, permits the hormone to attach to the cell surface *via* interactions with polyanionic glycosaminoglycans (GAGs) present on membrane glycoproteins such as heparan sulfate proteoglycan. Consistent with this theory was the significant reduction in the magnitude and duration of cAMP production in response to PTHrP_1-141_ in the presence of soluble heparin used as a decoy to prevent potential PTHrP_1-141_ and GAGs interactions (Fig. 3, *A* and *B*). The selective effect of heparin was verified by its lack of inhibitory action on cAMP induced by either PTH_1-34_ or PTHrP_1-36_ (Fig. 3*A*, and Table 1).

**Table 1:** Effect of heparin on cAMP production

Ligands	Control	Hep, 1 nM	*p* Value	Control	Hep, 10 nM	*p* Value
PTHrP_1-141_	100 ± 37 (33)	51 ± 30 (24)	< 0.0001	100 ± 37 (51)	38 ± 22 (52)	< 0.0001
PTHrP_1-36_	100 ± 26 (15)	129 ± 44 (9)	0.056	100 ± 47 (25)	92 ± 33 (39)	0.39
PTH_1-34_	100 ± 44 (43)	91 ± 31 (23)	0.38	100 ± 25 (39)	91 ± 35 (46)	0.18

The area under the curve (a.u.c) from data in Figure 3. Mean value ± SD of (N) experiments with *p* values determined by *t* test.

Abbreviations: Hep, heparin.

Collectively, these data prompt a reinterpretation of our previous understanding on how hormones act on the PTHR by providing compelling evidence that native PTHrP_1-141_ is biased toward sustained PTHR signaling *via* cAMP at the plasma membrane. The results support a model where PTHrP_1-141_ stabilizes an active receptor conformation that impairs βarr coupling and Gq signaling possibly through the interaction with GAG. Future experiments are needed for an extended characterization of PTHrP and GAG interaction as a possible means to reactivate recycled receptor by the cell surface–anchored hormone (Fig. 3*C*).

## Experimental procedures

Materials and methods are detailed in SI Appendix.

## Data availability

Source data are stored in Excel 2013 and will be deposited in the institutional repository of the University of Pittsburgh (http://d-scholarship.pitt.edu/).

## Supporting information

This article contains supporting information (1, 5, 6, 10, 13, 14, 15, 16, 17, 18).

## Conflict of interest

The authors declare that they have no conflicts of interest with the contents of this article.
