# Supplementary material for: Biased GPCR signaling by the native parathyroid hormone–related protein 1 to 141 relative to its N-terminal fragment 1 to 36
Source: J Biol Chem. 2022 Aug 4;298(9):102332. doi: 10.1016/j.jbc.2022.102332 (PMC9437850; doi:10.1016/j.jbc.2022.102332)
Supplement: Supporting information [file mmc1.docx]

**Supporting Information for**

Biased GPCR signaling by the native parathyroid hormone-related protein 1–141 relative to its N-terminal fragment 1–36

Karina A. Peña^1^, Alex D. White^1^, Sofya Savransky^1,2^, Ignacio Portales Castillo^3^, Frederic Jean-Alphonse^1^, Thomas J. Gardella^3^, Ieva Sutkeviciute^1^, and Jean-Pierre Vilardaga^1*^

^1^Department of Pharmacology and Chemical Biology, and ^2^Graduate Program in Molecular Pharmacology, University of Pittsburgh School of Medicine, Pittsburgh PA 15261, USA. ^3^Endocrine Unit, Massachusetts General Hospital and Harvard Medical School, Boston, MA 02114, USA.

*Corresponding author:

Jean-Pierre Vilardaga

([orcid.org/0000-0002-1217-1435](https://orcid.org/0000-0002-1217-1435))

**Email:**  [jpv@pitt.edu](mailto:jpv@pitt.edu)

**This file includes:**

Materials and Methods

Supplementary Information References

Supplementary Figure 1

Supplementary Figure 2

**Experimental Procedures**

**Peptides, chemicals, and plasmids.** Human PTH_1–34_, PTH_1-84_, and PTHrP_1–36_ were purchased from Bachem. Recombinant PTHrP_1–141_ (1) was a generous gift from Dr. T.J. Martin, or purified as described below, and synthetic PTHrP_1-141_ was synthesized as previously reported (2) and provided by Dr. S.J. Danishefsky. Peptides were resuspended in 10 mM acetic acid to make 1 mM stock solutions. Forskolin (#344270) was purchased from EMD-Millipore. DNA constructs encoding for PTHR^YFP^, PTHR^CFP^ and βarr-2^YFP^ were previously described by Vilardaga lab (3, 4). CMV-R-GECO1.2 was a gift from Robert Campbell (Addgene plasmid # 45494; http://n2t.net/addgene:45494; RRID:Addgene 45494). Heparin sodium salt was purchased from MedChem Express (#HY-17567A).

**Purification of recombinant PTHrP_1–141_ in physiological conditions.** GeneScript gene synthesis service was used to obtain the gene, subcloned to pEt21a vector, encoding PTHrP_1-141_, N-terminally fused to maltose-binding protein (MBP), followed by TEV cleavage site (ENLYFQ↓A), and C-terminally fused to Strep-Tag^®^II affinity tag (WSHPQFEK). The fusion was expressed in BL21(DE3) *E. coli* strain. Cells were grown in LB media supplemented with ampicillin and D-glucose (2 g/L), at 37°C and induced with 0.4 mM IPTG when OD_600_ reached 0.8. Following inductions, cells were grown at 37°C for 4 hours, then harvested and stored at -80°C until used. Cell pellet (worth of 1 L culture) was resuspended in 50 mL lysis buffer (25 mM Tris-HCl pH 8, 0.2 M NaCl, 1 mM EDTA, 0.1 mg/mL benzamidine, 0.002 mg/mL leupeptin, 0.007 mg/mL pepstatin A) and lysed by sonication. Cell lysate was spun down (30,000×g, 30 min) and supernatant was incubated with amylose resin (NEB), equilibrated in amylose wash buffer (25 mM Tris-HCl pH 8, 0.2 M NaCl, 1 mM EDTA). The resin was extensively washed, and the fusion protein was eluted with amylose elution buffer (25 mM Tris-HCl pH 8, 0.4 M NaCl, 1 mM MgCl_2_, 10 mM D-maltose). Amylose eluate after an overnight (at 4°C) treatment with salt-active nuclease (ArcticZymes), was subjected to a second affinity chromatography step using StrepTrap HP 5 mL column (GE Healthcare) equilibrated in 25 mM Tris-HCl, 0.4 M NaCl, and eluted in StrepTrap elution buffer (20 mM HEPES pH 7.4, 150 mM NaCl, 2.5 mM D-desthiobiotin). StrepTrap eluate was desalted by gel-filtration using Superose 6 10/300 column (GE Healthcare) equilibrated in SEC buffer (25 mM Tris-HCl, 150 mM NaCl, 0.5 mM EDTA, 100 μM TCEP). The desalted sample was treated overnight (on ice) with TEV protease added at 1:50 TEV:substrate mass ratio. The cleaved PTHrP_1-141_ was captured on StrepTrap HP column equilibrated in 20 mM HEPES pH 7.4, 150 mM NaCl, and eluted in StrepTrap elution buffer. The molecular weight of purified peptide was determined by MALDI TOF analysis at the Center for Molecular Analysis (Department of Chemistry, Carnegie Mellon University, Pittsburgh, USA), and bioactivity was measured as its ability to induce cAMP generation in HEK293 cells expressing PTHR (Supplementary Fig. 1).

**Cell culture***.* Cell culture reagents were obtained from Corning (CellGro). Human embryonic kidney cells (HEK293; ATCC, Georgetown, DC) stably expressing the recombinant human HA-tagged PTHR (5) were grown in DMEM supplemented with 5% FBS and 1% penicillin/streptomycin, at 37°C in a humidified atmosphere containing 5% CO_2_. For transient expression, cells were seeded in six-well plates and cultured for 24 h prior transfection with the appropriate cDNAs using Lipofectamine 3000 (Life Technologies) for 24 h. Subsequently, transfected cells were seeded on glass coverslips coated with poly-D-lysine in six-well plates and cultured for 24 hours before experiments.

**Radioligand receptor binding.** Competitive binding to the PTHR in G protein-dependent (R_G_) or G protein-independent (R_0_) states was assessed using membranes prepared from previously described COS-7 cells transiently expressing the rat PTHR (6). In brief, the R_0_ state was assayed in the presence of 10 mM GTPγS (to uncouple PTHR from G proteins) and using [^125^I]-Nle^8,21^,Y^34^-rPTH_1-34_ as tracer radioligand; the R_G_ state was assayed using membrane extracts prepared from cells expressing a negative-dominant Gα_S_ subunit and [^125^I]- Aib^1,3^,Y^15^,M-PTH_1-15_ as tracer radioligand. Reactions (230 µl) were assembled in 96-well vacuum filtration plates (MultiScreen; 0.65 μM, Durapore HV, Millipore Corp., Millford, MA) and contained membrane assay buffer (20 mM HEPES pH 7.4, 0.1 M NaCl, 3 mM MgSO_4_, 20% glycerol, 3 mg/ml bovine serum albumin (VWR #97068), 1X protease inhibitor cocktail (SIGMA cat# P8340) radioligand (~25,000 cpm/well), various concentrations of unlabeled ligand, and membranes (80 μg/ml), added last to start the reactions. Reactions were incubated at room temperature for 90 minutes and terminated by vacuum filtration followed by two rinses of the filters with membrane assay buffer; the filters were then detached and counted for gamma irradiation. Nonspecific binding was determined using 0.5 µM PTH_1–34_. Specific bound radioactivity was plotted *vs* ligand concentration using a three-parameter sigmoidal dose-response equation in GraphPad Prism version 9.0 (GraphPad Software, La Jolla California USA): $Y=Bottom+\frac{Top-Bottom}{1+{10}^{\left( X-Log[IC50] \right)}}$ with Y, specific binding; Top and Bottom, 100% and 0% specific radio-ligand binding, respectively; IC_50_, the half maximal inhibitory concentration of the unlabeled ligand; and X, Log[ligand].

**Time-course measurements of cAMP production, and PTHR recruitment of β-arrestin in single live cells**. cAMP production and β-arrestin recruitment were assessed using single cell FRET-based assays as previously described (7). In brief, cells were transiently transfected with the Epac1-CFP/YFP for measuring cAMP production, and PTHR C-terminally fused to CFP (PTHR-CFP) with βarr2-YFP for measuring arrestin recruitment. Measurements were performed on cells plated on poly-D-lysine coated glass coverslips and mounted in Attofluor cell chambers (Life Technologies) and maintained in HEPES buffer containing 150 mM NaCl, 20 mM HEPES, 2.5 mM KCl and 1 mM CaCl_2_, 0.1% BSA, pH 7.4. Cells were imaged on a Nikon Ti-E microscope equipped with an oil immersion 40X N.A 1.30 Plan Apo objective and a moving stage (Nikon Corporation). CFP and YFP were excited using a mercury lamp. Fluorescence emissions were filtered using a 480 ± 20 nm (for CFP) and 535 ± 15 nm (for YFP) filter set and collected simultaneously with a LUCAS EMCCD camera (Andor Technology) using a DualView 2 (Photometrics) with a beam splitter dichroic long pass (DCLP) of 505 nm. Fluorescence data were recorded from single cell using Nikon Element Software (Nikon Corporation). The FRET ratio for fluorescence emissions of CFP and YFP (F_YFP_/F_CFP_) for single cells was calculated and corrected for background, bleed-through, and photobleaching as previously described (8). Individual cells were perfused with buffer or with the ligand for the time indicated by the horizontal bar shown in plots. For heparin experiments, ligand and heparin were incubated at a 1:10 molar ratio 10 minutes prior cell stimulation.

**cAMP accumulation and ligand washout in multi-plate assays.** HEK293 cells stably expressing the Glosensor cAMP reporter and human PTHR were cultured in DMEM supplemented with 10% FBS. Cells were seeded into 96-well corning plates and used for cAMP assays after forming a confluent monolayer. Upon the removal of culture medium, intact cells in 96-well plates were incubated in CO_2_-independent medium containing D-luciferin (0.5 mM) for 20 minutes at room temperature. After this period, cells in each well were treated with peptides at various concentrations, and luminescence resulting from cAMP production was measured for 30 minutes on a BioTek Synergy 2 plate reader. The peak luminescence signaling usually appeared 14-20 minutes after peptide addition. For the washout experiments, cells preloaded with D-luciferin were treated with medium (vehicle) or agonists at various concentrations for 14 minutes. After this period, the medium in each well was removed and the cells were rinsed twice with CO_2_-independent medium to remove unbound ligand. After the addition of D-luciferin-containing fresh medium to each well, the luminescence was recorded for an additional 90 minutes. The concentration-response curves were fit to the data by using the following sigmoidal dose-response equation in GraphPad Prism version 9.0 (GraphPad Software, La Jolla California USA): $Y=Bottom+\frac{Top-Bottom}{1+{10}^{\left( Log[EC50]-X \right)\times n}}$ with Y, cAMP response; Bottom and Top, basal and maximal responses, respectively; EC_50_, concentration of ligand that gives a half response between Bottom and Top; X, Log[ligand]; and n, Hill slope.

**Photometric FRET recordings of PTHR activation in single cell assays.** FRET experiments were performed at the single cell level as previously described (7). In brief, cells grown on glass coverslips were maintained in a HEPES buffer containing 137 mM NaCl, 5 mM KCl, 1 mM MgCl_2_, 1 mM CaCl_2_, 20 mM HEPES, 0.1% (wt/vol) BSA, pH 7.4, at room temperature and placed on a Zeiss inverted microscope (Axiovert 200) equipped with oil immersion X60 and X100 objectives and a dual emission photometric system (Till Photonics, Germany). Cells expressing the intramolecular FRET-based PTHR sensor (9), PTHR^CFP/YFP^, were excited with light from a polychrome V (Till Photonics). The illumination time was set to 5–20 ms applied with a frequency of 5 Hz (200 ms). Individual cells were continuously perfused with the HEPES buffer without or with a ligand using a computer-assisted solenoid valve rapid superfusion device that permits rapid solution exchanges within 5 to 10 ms (ALA-VM8, ALA Scientific Instruments). The duration of ligand perfusion is indicated by a horizontal bar in time course plots. The emission fluorescence intensities were recorded at 535 ± 15 and 480 ± 20 nm (beam splitter dichroic long-pass 505 nm) upon excitation at 436 ± 10 nm (DCLP 460 nm). Fluorescent signals were detected by avalanche photodiodes, digitized using an Analog/Digital converter (Digidata1440A, Axon Instruments) and stored on a Personal Computer using Clampex version 10.4 software (Axon Instruments). The FRET ratio F_YFP_/F_CFP_ was corrected for the spillover of CFP into the 535-nm channel, the spillover of YFP into the 480-nm channel, and the direct YFP excitation, and changes in fluorescence emissions due to photobleaching were systematically subtracted. Changes in the FRET ratio are normalized to the initial value at t = 0 s. The decrease of the FRET ratio was fitted by a mono-exponential decay $A\times(1-e^{-t/\tau}$, where *t* is the time (s), *τ* is the time constant (s) and *A* is the magnitude of the signal.

**Intracellular Ca^2+^ release in single cell assays.** Time courses of intracellular calcium [iCa^2+^] changes were measured in HEK-PTHR cells expressing the Red-GECO1.2 fluorescent Ca^2+^ sensor. Transfected cells were seeded on poly-D-lysine coated coverslips and mounted in Attofluor cell chambers (Life Technologies). Cells were incubated with a buffer containing 150 mM NaCl, 20 mM HEPES, 2.5 mM KCl and 1 mM CaCl_2_, 0.1% BSA, pH 7.4, and imaged on a Nikon Ti-E microscope (Nikon) equipped with a Z-driven piezo motor and a heated stage. Imaging was performed using Nikon A1 confocal unit, through a 60 × 1.45 N.A. plan-apo objective (Nikon). R-GECO1.2 sensor was excited with 561-nm laser (Melles Griot). Single cells were imaged at 6 seconds intervals and stimulated with the indicated ligands 2 minutes after recording started. Fluorescent intensity for single cells and background were calculated using Nikon Element Software (Nikon Corporation).

**Receptor internalization and recycling in single cell-based assays.** PTHR internalization and recycling was quantitated by single-cell imaging of human PTHR N-terminally tagged with a pH-sensitive GFP variant, superecliptic pHluorin (PTHR^SEP^) as previously described (10) using a Nikon A1 confocal microscope. HEK293 cells stably expressing PTHR^SEP^ were seeded on glass coverslips coated with poly-D-lysine (Sigma, P6407) for 24 hours. Experiments were carried out at 37°C in HEPES buffer used for cAMP experiments. Cells were stimulated by 100 nM ligand for 3 minutes then washed out to allow recycling. Images were acquired every 30 seconds*.* The change in fluorescence Δ*F* = F_0_ – *F* was normalized as $\frac{\Delta F}{F_{0}}$ with F_0,_ initial fluorescence at t = 0 min and in the absence of the ligand, and *F*, fluorescence at t > 0 min. The initial fluorescence was set to 100%.

**Statistical analysis***.* Data were processed using Origin version 8.0 (OriginLab Corporation), and GraphPad Prism version 7.0, 8.0 or 9.0 (GraphPad Software, La Jolla California USA). Data are expressed as mean ± SD or SEM. Binding data from concentration–response assays were analyzed by using a sigmoidal dose–response model with variable slope. Statistical analyses for data involving one independent variable (either cAMP, iCa^2+,^ or receptor activation kinetics) and three groups (PTH_1-34_, PTHrP_1-36_, and PTHrP_1-141_) were performed using one-way ANOVA with Tukey post-hoc test.

**Supplementary Information References**

1. Hammonds RG, Jr.*, et al.* (1989) Purification and characterization of recombinant human parathyroid hormone-related protein. *The Journal of biological chemistry* 264(25):14806-14811.

2. Li J*, et al.* (2012) Chemistry as an expanding resource in protein science: fully synthetic and fully active human parathyroid hormone-related protein (1-141). *Angew Chem Int Ed Engl* 51(49):12263-12267.

3. Feinstein TN*, et al.* (2011) Retromer terminates the generation of cAMP by internalized PTH receptors. *Nature chemical biology* 7(5):278-284.

4. Wehbi VL*, et al.* (2013) Noncanonical GPCR signaling arising from a PTH receptor-arrestin-Gbetagamma complex. *Proc Natl Acad Sci U S A* 110(4):1530-1535.

5. Castro M*, et al.* (2002) Dual regulation of the parathyroid hormone (PTH)/PTH-related peptide receptor signaling by protein kinase C and beta-arrestins. *Endocrinology* 143(10):3854-3865.

6. Dean T, Vilardaga JP, Potts JT, Jr., & Gardella TJ (2008) Altered selectivity of parathyroid hormone (PTH) and PTH-related protein (PTHrP) for distinct conformations of the PTH/PTHrP receptor. *Molecular endocrinology* 22(1):156-166.

7. Gidon A*, et al.* (2014) Endosomal GPCR signaling turned off by negative feedback actions of PKA and v-ATPase. *Nature chemical biology* 10(9):707-709.

8. Vilardaga JP (2011) Studying ligand efficacy at G protein-coupled receptors using FRET. *Methods Mol Biol* 756:133-148.

9. Vilardaga JP, Bunemann M, Krasel C, Castro M, & Lohse MJ (2003) Measurement of the millisecond activation switch of G protein-coupled receptors in living cells. *Nat Biotechnol* 21(7):807-812.

10. McGarvey JC*, et al.* (2016) Actin-Sorting Nexin 27 (SNX27)-Retromer Complex Mediates Rapid Parathyroid Hormone Receptor Recycling. *The Journal of biological chemistry* 291(21):10986-11002.

**Supplementary Figure 1. Purification of recombinant PTHrP_1-141_.** (*A*) Amino acid sequence of PTHrP_1-141_ N-terminally fused to MBP; arrow indicates the TEV cleavage site. (*B*) SDS-PAGE analysis of PTHrP_1-141_ purification: *left*, lanes 1, 2, 3 show samples before desalting (fusion protein alone, theoretical MW 58.4 kDa), desalted before TEV cleavage, and TEV-treated sample, respectively; *right*, final purified PTHrP_1-141_ (theoretical average MW 17188.16 Da) The gels were spliced as indicated by black boxes to show only relevant lanes. (*C*) MALDI-TOF mass spectrum of final purified PTHrP_1-141_. (*D*) Time courses of cAMP in single HEK293 cells stably expressing the recombinant human PTHR. Individual cells perfused with buffer or 1 nM ligand (horizontal bar). Data are the mean ± SEM of *n* = 12 for recombinant PTHrP_1-36_), and *n* = 5 for synthetic PTHrP_1-141_).

**Supplementary figure 2**. Time courses of internalization and recycling of PTHR tagged with super-ecliptic pHluorin (PTHR^SEP^) in response to 100 nM ligand measured by time-lapse confocal microscopy in single cells. Data are means ± SEM from *n* = 12 (PTH_1-34_) *n* = 51 (PTHrP_1-141_) and n = 21 (PTHrP_1-36_) cells.
